# Return to play is reliable, but market value declines after proximal hamstring repair in elite soccer players

**DOI:** 10.1002/jeo2.70775

**Published:** 2026-06-22

**Authors:** Gianluca Ciccarelli, Edoardo Monaco, Valerio Nasso, Daniele Mazza, Riccardo D'Ambrosi, Pierfrancesco Orlandi, Alessandro Annibaldi, Alessandro Carrozzo

**Affiliations:** ^1^ Department of Orthopedic Surgery and Traumatology, AOU Sant'Andrea, School of Medicine and Psychology University of Rome ‘La Sapienza’ Rome Italy; ^2^ IRCCS Ospedale Galeazzi—Sant'Ambrogio Milan Italy; ^3^ Dipartimento di Scienze Biomediche per la Salute Università degli Studi di Milano Milan Italy; ^4^ Dipartimento di Scienze della Vita, della Salute e delle Professioni Sanitarie Università degli Studi ‘Link Campus University’ Rome Italy

**Keywords:** elite soccer players, market value, proximal hamstring injury, return to play, surgical repair

## Abstract

**Purpose:**

The purpose of this study was to quantify return‐to‐play (RTP) time, post‐operative performance and market‐value trends after proximal hamstring repair in top‐tier European league soccer players. It was hypothesized that surgery would achieve a high RTP rate but be accompanied by reductions in player performance and valuation.

**Methods:**

Male first‐team players from the five major European leagues who underwent surgical repair of proximal hamstring ruptures between 2019 and 2022 were identified collaboratively with the InjuryMechanisms platform. Publicly available data (Transfermarkt) was accessed to extract demographics and season‐by‐season metrics for two seasons before and two seasons after injury. Outcomes were RTP time, matches missed, percentage of minutes played per season (MPS), goals, assists and market value. Non‐parametric Friedman test with Dunn–Bonferroni post hoc test assessed longitudinal changes.

**Results:**

Twenty players (mean age, 25.3 ± 4.4 years) met the inclusion criteria. All returned to professional competition without reoperation. Mean RTP was 160 ± 76 days, with 21.5 ± 11.5 matches missed. Goals and assists were unchanged across seasons (*p* > 0.05). MPS showed a non‐significant reduction from 50.2 ± 15.5% pre‐injury to 34.5 ± 16.3% in the first post‐operative season and partially recovered to 43.0 ± 23.9% in the second season. Mean market value significantly declined from €22.1 ± 36.7 and €20.4 ± 34.4 million in the two pre‐injury seasons to €15.2 ± 27.4 million two seasons post (*p* < 0.001).

**Conclusion:**

Surgical repair of proximal hamstring rupture allowed reliable return to competition in elite soccer players, with preserved performance. However, players experienced a transient, non‐significant reduction in competitive involvement during the first post‐operative season and market value significantly declined despite favourable performance outcomes.

**Level of Evidence:**

Level IV.

## Graphical abstract

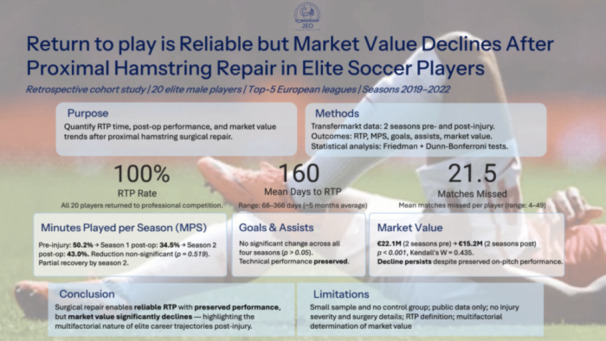

**Abbreviations**
BMI: body mass index
IQR: interquartile range
MPS: minutes played per season
MRI: magnetic resonance imaging
PHI: proximal hamstring injuries
RTP: return to play
SD: standard deviation

## INTRODUCTION

Hamstring injuries are among the most common and impactful musculoskeletal injuries in athletes, often resulting in prolonged absence from competition, significant reduction in performance and potentially athlete's career ending [3].

Despite increasing attention to the diagnosis and management of hamstring ruptures, as well as the introduction of prevention protocols, the incidence and burden of hamstring injuries during training and match play among professional soccer players have increased significantly. During recent seasons, these injuries accounted for 14% of the total injury‐related lay‐off duration, ranging from 12% in 2001/2002 to 24% in 2021/2022 [11]. The majority of hamstring injuries involve proximal hamstring injuries (PHI), with the biceps femoris most commonly injured, in particular at the musculotendinous junction [1, 2]. The severity of the injury can vary from a minor muscle injury, which represents 25%–30% of all muscle strains, to a complete avulsion of three tendinous insertions on the ischial tuberosity [5].

The management of the PHI depends on the site and severity of the tear, and currently, there is no definitive consensus on the most effective treatment approach [6, 7, 9]. Surgical repair has demonstrated good functional outcomes and high return to sport rate, both for complete and partial injuries, and particularly in an acute setting [19, 22], while delayed treatment, and some cases of non‐operative treatment, often resulted in functional deficits and poorer outcomes [16].

Beyond the consequences for individual player performance, PHI may also negatively affect club performance and financial outcomes. Reduced player availability may compromise competitive results, while the injury's impact on players' careers can lower their market value [14, 29].

The aim of this study was to evaluate the performance and financial impact of surgically treated PHI on a cohort of professional soccer players.

The hypothesis was that surgical treatment of PHI in professional soccer players would lead to a high rate of return to sport, but that can be associated with a significant decline in post‐operative athletic performance and market value when compared to pre‐injury levels.

## METHODS

In this retrospective study, the authors identified male professional soccer players undergoing surgery for proximal hamstring rupture between 2019 and 2022 in the five major European championships (Italian, English, German, French and Spanish). Case identification was conducted in July 2025. During the screening process, a collaborative effort was undertaken with the InjuryMechanisms page on X.com (@IMechanisms), which contributed to the identification and verification of relevant cases through systematic monitoring of publicly available match reports, press releases and video analyses. The study included only players who were members of the first senior team at the time of injury and had available data for the two seasons preceding and the two seasons following the injury. Demographic and clinical data, including age, body mass index (BMI), affected limb, playing role, return‐to‐play (RTP) time as well as the number of missed matches, appearances, total minutes played, total playable minutes, the percentage of minutes played per season (MPS) before and after surgery and the number of goals and assists per season, were obtained from the publicly accessible media‐based database Transfermarkt (https://www.transfermarkt.com/). Finally, player's market value trend before and after injury was analysed. In cases of players with incomplete or missing information, supplementary data were gathered from social team websites, press releases and other additional publicly available online sources, otherwise they were excluded. Ethics approval was not required for this study, as it was based exclusively on publicly available online sources and did not involve access to medical records, imaging data or any sensitive patient‐level information. The RTP time was defined as the time from the hamstring injury to the player's first official match. The MPS were calculated as the proportion of minutes played relative to the total available match minutes in the two seasons preceding the injury and for the two seasons following the injury.

### Statistical analysis

All analyses were performed with SPSS® Statistics software (Version 27.0; IBM SPSS). Descriptive data were analysed for the entire patient cohort. Descriptive data analyses were conducted depending on the nature of the considered criteria. For quantitative data, this included the number of observed values, mean, standard deviation (SD), median and interquartile range (IQR). For qualitative data, this included the number of observed and missing values and the number and percentage of patients per class. The level of statistical significance was set at *p* < 0.05. Comparisons between categorical variables were assessed with a *χ* ^2^ or Fisher's exact test. Comparison between quantitative variables was assessed with non‐parametric Friedman test. Due to the limited sample size, exact *p* values were interpreted to ensure robustness, and Kendall's *W* was calculated to assess effect size. The post hoc comparisons were assessed by the Dunn–Bonferroni test. The normality of variables was assessed with a Shapiro–Wilk test.

## RESULTS

During the study period, 21 elite male soccer players sustained a PHI requiring surgical treatment. Moreover, 20 players were included in this study, while 1 player was excluded due to insufficient available data on demographic and clinical outcomes.

Mean age at the time of the injury was 25.3 ± 4.4 years, ranging from 19 to 34 years.

The majority of injuries occurred in the Premier League (70%), with a relatively balanced distribution across defenders, midfielders and forwards.

Demographic data are shown in more detail in Table 1.

**Table 1:** Demographic of the study population.

	Total population *n* = 20
Age, years	25.3 ± 4.4 (19–34)
BMI	22.5 ± 1.8 (19.7–26.1)
Side, *n* (%)	
Left	3 (15)
Right	12 (60)
Not reported	5 (25)
Position	
Forward	6 (30)
Midfielder	7 (35)
Defender	7 (35)
Leagues	
Premier League	14 (70)
Ligue 1	1 (5)
Serie A	1 (5)
LaLiga	3 (15)
Bundesliga	1 (3)

*Note*: Data are presented as mean ± SD (range) or *n* (%).

Abbreviations: BMI, body mass index; SD, standard deviation.

The mean time to RTP was 160.1 ± 76.2 days (range, 68–366), with an average of 21.45 ± 11.5 matches missed (range, 4–49). None of the players included in the study required revision surgery or additional procedures related to the hamstrings over the two post‐injury seasons.

**Figure 1:**
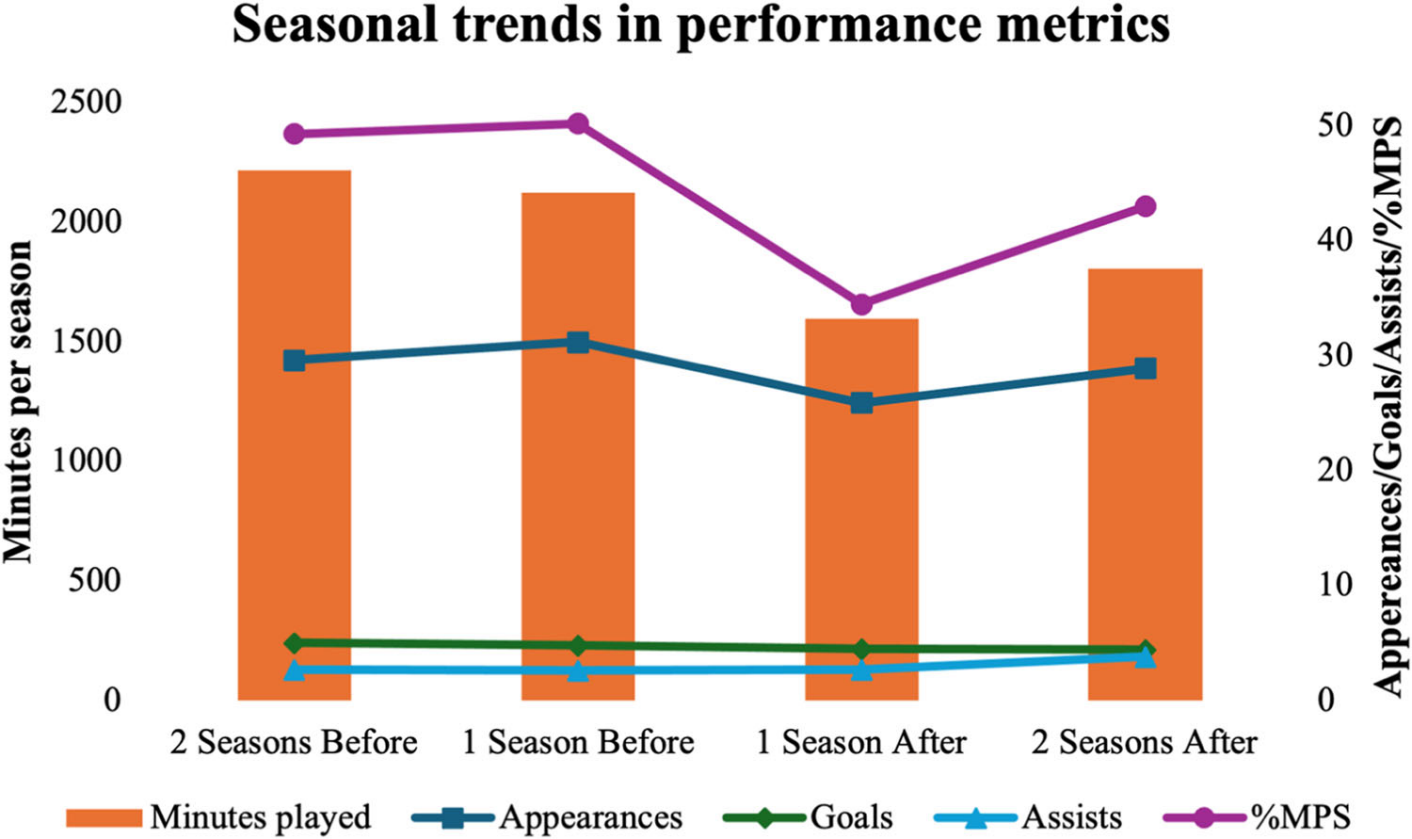
Seasonal trends in performance metrics pre‐ and post‐injury outcomes. MPS, minutes per season.

There were no statistically significant differences across the seasons in terms of appearances, total minutes played, MPS, goals or assists (Table 2 and Figure 1).

**Table 2:** Seasonal trends in performance metrics pre‐ and post‐injury outcomes.

	Appearances mean ± SD	Minutes played mean ± SD	Goals mean ± SD; median (IQR)	Assists mean ± SD; median (IQR)	Percentage of MPS mean% ± SD
Two seasons before injury	29.6 ± 13.1	2217.4 ± 1265.4	5 ± 9.4; 2.5 (1–5)	2.7 ± 3.3; 1.5 (0.25–6.25)	49.3 ± 30.0
One season before injury	31.2 ± 8.1	2123.8 ± 740.1	4.8 ± 6.1; 2 (0–5)	2.6 ± 2.6; 2 (0–4)	50.2 ± 15.5
One season after injury	25.9 ± 11.5	1596.3 ± 973.1	4.5 ± 7.9; 2 (1–7)	2.7 ± 4; 1 (1–5)	34.5 ± 16.3
Two seasons after injury	28.9 ± 12.3	1808.2 ± 1104.9	4.4 ± 6.4; 2.5 (1–3.75)	3.8 ± 4.3; 1 (0–3.75)	43.0 ± 23.9
Overall *p* value	0.702	0.559	0.881	0.230	0.519

*Note*: Results are reported as mean ± SD. For skewed variables, the median and IQR are reported.

Abbreviations: IQR, interquartile range; MPS, minutes per season; SD, standard deviation.

However, a significant reduction in player market value was observed, with the Friedman test revealing a statistically significant trend (exact *p* value < 0.001, Kendall's *W* = 0.435).

**Figure 2:**
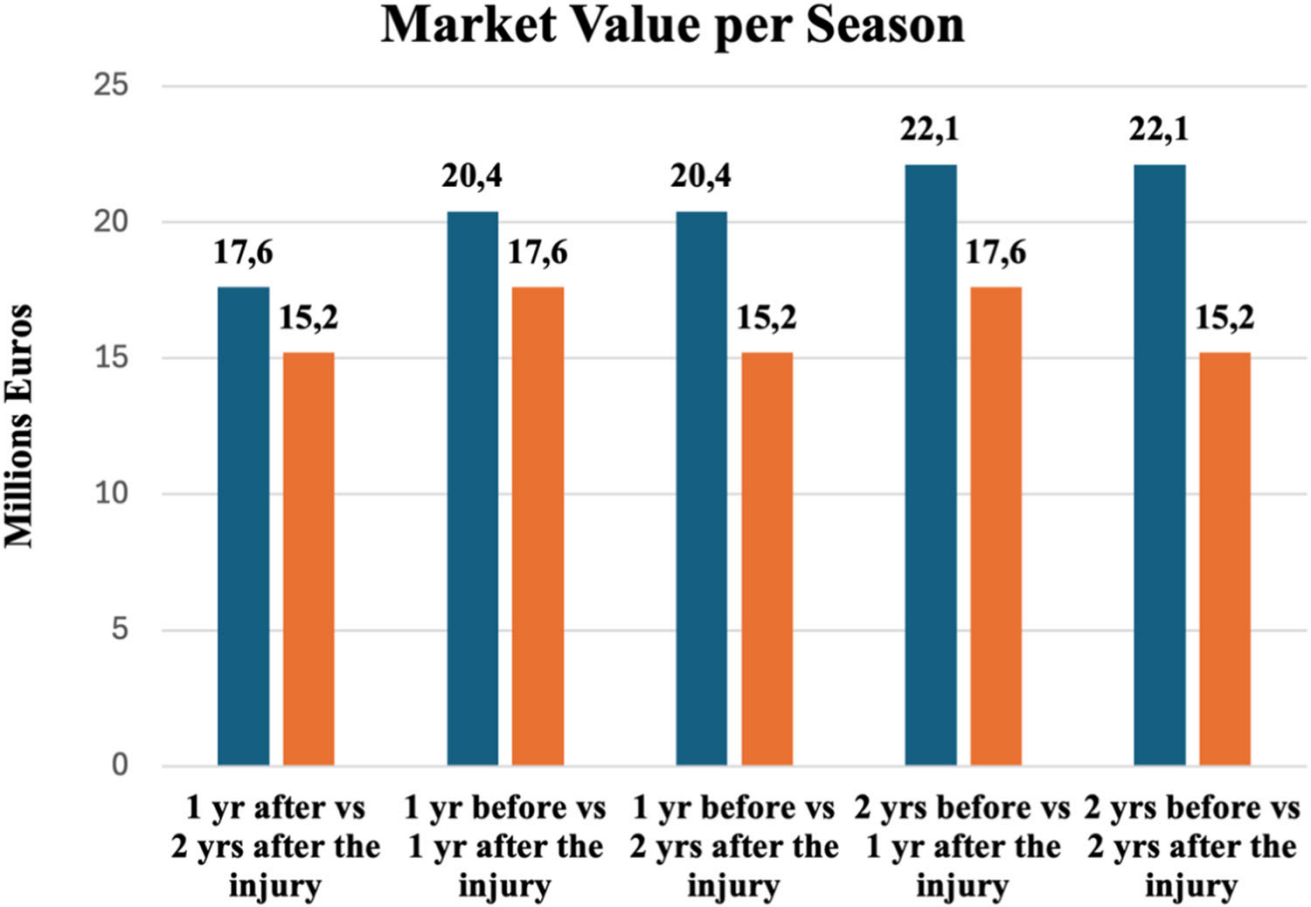
Comparison of player market value before and after hamstring injury.

Mean market value declined from €22.1 ± €36.7 million two seasons before the injury to €15.2 ± €27.3 million in the second season after the injury (*p* < 0.001), and from €20.4 ± €34.4 million in the season before the injury to €15.2 ± €27.4 million in the second season after the injury (*p* < 0.001). Comparison between market value across the seasons is resumed in Table 3 and shown graphically in Figure 2.

**Table 3:** Comparison of player market value before and after hamstring injury.

	Market value per season		
Comparison	Mean ± SD	Median (IQR)	Adjusted *p* value
One season after injury versus	17.6 ± 28.2	9 (4–16.5)	0.165
Two seasons after the injury	15.2 ± 27.3	7.25 (3–12)	
One season before the injury versus	20.4 ± 34.4	11.5 (5–17.25)	0.193
One season after the injury	17.6 ± 28.2	9 (4–16.5)	
One season before the injury versus	20.4 ± 34.4	11.5 (5–17.25)	**<0.001**
Two seasons after the injury	15.2 ± 27.4	7.25 (3–12)	
Two seasons before the injury versus	22.1 ± 36.7	11 (6.25–18)	0.455
One season after the injury	17.6 ± 28.2	9 (4–16.5)	
Two seasons before the injury versus	22.1 ± 36.7	11 (6.25–18)	**<0.001**
Two seasons after the injury	15.2 ± 27.4	7.25 (3–12)	

*Note*: Values are reported in million euros as mean ± SD and median (IQR). Bold values indicate statistically significant at *p* < 0.05.

Abbreviations: IQR, interquartile range; MPS, minutes per season; SD, standard deviation.

## DISCUSSION

The main finding of this study was that surgical repair of PHI enabled elite soccer players to return to competition within about 5 months. Contrary to initial expectations, although match exposure tended to decrease during the first postoperative season, this reduction was not statistically significant, and technical performance indicators, such as goals and assists, were preserved. In contrast, player market value declined significantly between the pre‐ and post‐injury seasons.

Hamstring injuries are among the most common and impactful musculoskeletal injuries in soccer players. These injuries can occur in both older athletes, potentially contributing to early retirement and younger athletes, in whom they may cause a potential deterioration in career development, accounting for approximately one‐third of total time‐loss [10, 15, 31]. Consistently, in the current study, the mean age of the soccer players analysed was 25.3 ± 4.4 years, ranging from 19 to 34 years, with a mean of 21 missed matches. However, no retirement from professional sport has been described in the two seasons following the injury. Injuries were distributed relatively evenly across playing positions; however, a higher incidence was observed in the Premier League, which is likely to reflect the greater physical demands of this competition [27, 35], thus aligning with previous literature, which identifies level of activity and match workload as key risk factors for PHI [10, 13, 17].

To date, the optimal management of PHI remains still widely debated, largely depending on the site and severity of the tear [20, 34]. In recent years, surgical intervention has gained popularity, particularly among athletes, in whom factors such as injury‐related layoff time, time to RTP and recurrence rates are critical and should be minimized [21]. According to the London International Hamstring Consensus [30], surgical repair is specifically indicated in cases of gapping and loss of tension at the injury site, symptomatic displaced bony avulsions and proximal free tendon injuries associated with functional compromise that is refractory to non‐operative treatment, preferably performed in the acute setting [6]. Several authors have reported high return to sport rates and favourable functional outcomes following surgical intervention. In the study of Ayuob et al., 64 proximal biceps femoris musculotendinous junction injuries were surgically repaired in high‐level athletes, with 100% returning to preinjury level of sport at a mean ± SD time of 13.4 ± 5.1 weeks [4]. Castelli et al. reported a mean return‐to‐sport time of 7.0 months (7.0–12.0) in athletes under 25 years of age and 5.0 months (3.3–8.0) in those over 25 after surgical repair of proximal avulsion of the hamstrings [8]. Finally, Lempainen et al. described excellent or good results also after surgical repair of proximal partial hamstring tear, allowing the athlete to reduce the time away from sports activities due to persistent pain and weakness [22]. Aligning with these results, in the current analysis, 100% of athletes were able to return to sport after surgical repair of PHI, with a mean return‐to‐sport time of 160.1 ± 76.2 days (range, 68–366).

Although all players were able to RTP, this metric alone does not provide a clear insight into players' performance following their return to competition. In this study, multiple performance metrics were examined. Match exposure appeared to decrease during the first season following injury; however, the reduction in the percentage of MPS between the pre‐ and post‐injury seasons (50.2 ± 15.5 vs. 34.5 ± 16.3, respectively) did not reach statistical significance. A similar trend has also been documented in literature [25, 26] following other types of injuries and may be attributed to several factors, including physical limitations [33] as well as psychological aspects [12], which could prevent players from being fully prepared to return to competition. Nevertheless, compared to the first post‐operative season, MPS increased in the second (43.0 ± 23.9 vs. 34.5 ± 16.3) and other performance metrics, such as goals and assists, were preserved when comparing the two seasons before and after the injury, thus suggesting that surgical repair of PHI may promote a stable and sustained restoration of performance. Biologically, the favourable outcomes associated with surgical repair may be explained by its ability to promote more effective healing of the injury. Indeed, as reported by van der Made et al. [24], magnetic resonance imaging (MRI) findings showed tendon continuity restoration in only 52% of non‐operatively treated patients, with a considerable proportion exhibiting varying degrees of fatty infiltration. Similar to what is observed in rotator cuff injuries, these structural changes may result in reduced muscle function and a progressive decline in athletic performance over time. Although the results of this study were positive, these should be interpreted with caution, as performance metrics are strongly influenced by playing position, timing of injury within the season and other external factors. In addition, due to the small sample size, subgroup analyses could not be performed.

As well as on the performance of the individual athlete, it is also well demonstrated that hamstring lesions can have a negative impact on the entire club, affecting both competitive results and the club's financial situation [28, 29]. As described in the study by Hägglund et al. [14], a lower injury burden and higher match availability were associated with an increased number of points earned per league match, as well as with a higher Union of European Football Association (UEFA) Season Club Coefficient, reflecting greater success in the UEFA Champions League or Europa League and finally greater financial gains too. In addition to poor competitive results, financial losses for soccer clubs are also determined by a decrease in the players' market value of the team. This reduction is a normal phenomenon influenced by many factors such as the occurrence of injuries, age, physiological decline in performance over the years, contract status, broader economic fluctuations and many other external factors [32]. In the current study, despite favourable clinical outcomes, the average players' market value still significantly declined from 22.1 ± 36.7 million euros before injury to 15.2 ± 27.4 million euros after injury. Due to the absence of a control group of healthy players, it is not possible to determine whether this finding reflects the normal age‐related decline in performance or if the PHI played a primary role. However, the observation that athletic performance remained largely unchanged following surgery may have helped mitigate this effect, limiting its impact on players' market value and reducing potential financial losses for clubs.

From a practical perspective, these findings suggest that, although surgical repair of a proximal hamstring rupture enables a reliable return to professional competition, full reintegration into competitive play, as reflected by match exposure, may require more than one season. Furthermore, traditional performance metrics such as goals and assists appear to be unaffected, while metrics on the availability of the players are a more sensitive indicator of post‐injury impact. Finally, favourable clinical recovery does not necessarily lead to preservation of market value, highlighting the multifactorial nature of career trajectories in elite football.

Several limitations of this study should be considered. First, it was a retrospective study with a small sample size, which did not allow for subgroup analyses, and the absence of a matched control group precluded comparisons with conservatively treated players or healthy players in terms of performance and career outcomes. Secondly, data were collected from publicly available online sources. As reported in the recent literature [18], this may have underestimated the true incidence of injuries; consequently, some injured players may not have been included in the present analysis. Moreover, in the absence of access to medical records or imaging data, detailed information regarding injury characteristics, such as rupture pattern (complete vs. partial), number of tendons involved, degree of retraction, timing of surgery (acute vs. delayed), surgical techniques and club‐specific rehabilitation protocols, was unavailable. These factors may influence recovery patterns and post‐injury performance, and analyses stratified by such variables could provide additional insights. However, given the lack of a centralized injury database for European professional soccer comparable to those available in the National Football League (NFL) or the National Basketball Association (NBA), publicly accessible sources represent the most feasible and independent option and have been successfully used in previous studies [23, 25].

Furthermore, RTP was defined as participation in official professional matches and did not account for return to training. As a result, some players may have been medically cleared and fully recovered but not selected for match play. This definition may also have influenced the number of matches missed, which is additionally affected by the timing of injury within the competitive season. Finally, market value was included as an outcome measure, but it is known that this can be influenced by multiple external and career‐related factors (e.g., age, contract duration, transfers, club and league context and global market dynamics), and separating the isolated effect of injury or surgery was not feasible within a small retrospective cohort.

Despite these limitations, the present study was designed primarily to describe the outcomes of surgical repair of PHI and RTP in European professional soccer players, providing preliminary insights that may help physicians, athletes and clubs in setting realistic expectations following this injury. Further studies investigating additional variables are warranted to confirm and expand these findings.

## CONCLUSION

Surgical repair of proximal hamstring rupture allowed reliable return to competition in elite soccer players, with preserved performance. However, players experienced a transient, non‐significant reduction in competitive involvement during the first post‐operative season and market value significantly declined despite favourable performance outcomes.

## AUTHOR CONTRIBUTIONS

**Gianluca Ciccarelli**: Data acquisition; manuscript writing. **Edoardo Monaco**: Conceptualization; manuscript editing. **Valerio Nasso**: Data analysis. **Daniele Mazza**: Manuscript editing. **Riccardo D'Ambrosi**: Data analysis. **Pierfrancesco Orlandi**: Data acquisition**. Alessandro Annibaldi**: Manuscript editing. **Alessandro Carrozzo**: Data acquisition; manuscript editing.

## FUNDING INFORMATION

The authors have no funding to report.

## CONFLICT OF INTEREST STATEMENT

Edoardo Monaco is consultant for Arthrex. The remaining authors declare no conflict of interest.

## ETHICS STATEMENT

The authors have nothing to report.

## Data Availability

All data supporting the findings of this study are available within the paper and its Supporting Information.

## References

[1] Allahabadi S , Salazar LM , Obioha OA , Fenn TW , Chahla J , Nho SJ . Hamstring injuries: a current concepts review: evaluation, nonoperative treatment, and surgical decision making. Am J Sports Med. 2024;52(3):832–844. 37092718 10.1177/03635465231164931

[2] Arner JW , Rothrauff B , Bradley JP . Hamstring injuries in athletes: anatomy, pathology, and treatment. J Am Acad Orthop Surg. 2025;33(13):e703–e714. 39928857 10.5435/JAAOS-D-24-01162

[3] Askling CM , Tengvar M , Saartok T , Thorstensson A . Proximal hamstring strains of stretching type in different sports: injury situations, clinical and magnetic resonance imaging characteristics, and return to sport. Am J Sports Med. 2008;36(9):1799–1804. 18448581 10.1177/0363546508315892

[4] Ayuob A , Kayani B , Haddad FS . Musculotendinous junction injuries of the proximal biceps femoris: a prospective study of 64 patients treated surgically. Am J Sports Med. 2020;48(8):1974–1982. 32603235 10.1177/0363546520926999

[5] Bertiche P , Mohtadi N , Chan D , Hölmich P . Proximal hamstring tendon avulsion: state of the art. J ISAKOS. 2021;6(4):237–246. 34272300 10.1136/jisakos-2019-000420

[6] Bodendorfer BM , Curley AJ , Kotler JA , Ryan JM , Jejurikar NS , Kumar A , et al. Outcomes after operative and nonoperative treatment of proximal hamstring avulsions: a systematic review and meta‐analysis. Am J Sports Med. 2018;46(11):2798–2808. 29016194 10.1177/0363546517732526

[7] Buckwalter J , Westermann R , Amendola A . Complete proximal hamstring avulsions: is there a role for conservative management? A systematic review of acute repairs and non‐operative management. J ISAKOS. 2017;2(1):31–35.

[8] Castelli A , Parenti M , Tirone G , Spera M , Azzola F , Zanon G , et al. Proximal avulsion of the hamstring in young athlete patients: a case series and review of literature. Eur J Orthop Surg Traumatol. 2024;34(8):4139–4147. 39414664 10.1007/s00590-024-04096-1 PMC11519243

[9] Chang JS , Kayani B , Plastow R , Singh S , Magan A , Haddad FS . Management of hamstring injuries: current concepts review. Bone Joint J. 2020;102(10):1281–1288. 32993323 10.1302/0301-620X.102B10.BJJ-2020-1210.R1

[10] Della Villa F , Massa B , Bortolami A , Nanni G , Olmo J , Buckthorpe M . Injury mechanisms and situational patterns of severe lower limb muscle injuries in male professional football (soccer) players: a systematic video analysis study on 103 cases. Br J Sports Med. 2023;57(24):1550–1558. 37898508 10.1136/bjsports-2023-106850

[11] Ekstrand J , Bengtsson H , Waldén M , Davison M , Khan KM , Hägglund M . Hamstring injury rates have increased during recent seasons and now constitute 24% of all injuries in men's professional football: the UEFA Elite Club Injury Study from 2001/02 to 2021/22. Br J Sports Med. 2023;57(5):292–298. 10.1136/bjsports-2021-105407 PMC9985757 36588400

[12] Glazer DD . Development and preliminary validation of the Injury‐Psychological Readiness to Return to Sport (I‐PRRS) scale. J Athl Train. 2009;44(2):185–189. 19295964 10.4085/1062-6050-44.2.185 PMC2657021

[13] Guitart‐Trench M , Sanchez‐Sanchez J , Valle X , Garcia‐Unanue J , Cos F , Alonso‐Callejo A , et al. Does accumulated physical load in different time windows affect hamstring injuries in elite football players? Res Sports Med. 2025;33(4):427–439. 39976375 10.1080/15438627.2025.2468799

[14] Hägglund M , Waldén M , Magnusson H , Kristenson K , Bengtsson H , Ekstrand J . Injuries affect team performance negatively in professional football: an 11‐year follow‐up of the UEFA Champions League injury study. Br J Sports Med. 2013;47(12):738–742. 23645832 10.1136/bjsports-2013-092215

[15] Hall ECR , Larruskain J , Gil SM , Lekue JA , Baumert P , Rienzi E , et al. Injury risk is greater in physically mature versus biologically younger male soccer players from academies in different countries. Phys Ther Sport. 2022;55:111–118. 35325670 10.1016/j.ptsp.2022.03.006

[16] Hofmann KJ , Paggi A , Connors D , Miller SL . Complete avulsion of the proximal hamstring insertion: functional outcomes after nonsurgical treatment. J Bone Joint Surg. 2014;96(12):1022–1025. 24951738 10.2106/JBJS.M.01074

[17] den Hollander S , Kerkhoffs G , Gouttebarge V . The impact of match workload and international travel on injuries in professional men's football. Sports. 2024;12:212. 39195588 10.3390/sports12080212 PMC11360389

[18] Inclan PM , Chang PS , Mack CD , Solomon GS , Brophy RH , Hinton RY , et al. Validity of research based on public data in sports medicine: a quantitative assessment of anterior cruciate ligament injuries in the National Football League. Am J Sports Med. 2022;50(6):1717–1726. 34166138 10.1177/03635465211015435

[19] Lefèvre N , Moussa MK , Alban Bouché P , Valentin E , Gerometta A , Khiami F , et al. Improved outcomes of proximal hamstring avulsion surgery in patients both under and over 50 years, with greater gains in the younger group: a matched comparative study of the PHAS cohort. Knee Surg Sports Traumatol Arthrosc. 2025;33(5):1853–1862. 39844676 10.1002/ksa.12596

[20] Lempainen L , Banke IJ , Johansson K , Brucker PU , Sarimo J , Orava S , et al. Clinical principles in the management of hamstring injuries. Knee Surg Sports Traumatol Arthrosc. 2015;23(8):2449–2456. 24556933 10.1007/s00167-014-2912-x

[21] Lempainen L , Kosola J , Pruna R , Sinikumpu JJ , Valle X , Heinonen O , et al. Tears of biceps femoris, semimembranosus, and semitendinosus are not equal—a new individual muscle‐tendon concept in athletes. Scand J Surg. 2021;110(4):483–491. 33612019 10.1177/1457496920984274 PMC8688976

[22] Lempainen L , Sarimo J , Heikkilä J , Mattila K , Orava S . Surgical treatment of partial tears of the proximal origin of the hamstring muscles. Br J Sports Med. 2006;40(8):688–691. 16790482 10.1136/bjsm.2006.028191 PMC2579455

[23] Locks R , Utsunomiya H , Briggs KK , McNamara S , Chahla J , Philippon MJ . Return to play after hip arthroscopic surgery for femoroacetabular impingement in professional soccer players. Am J Sports Med. 2018;46(2):273–279. 29135269 10.1177/0363546517738741

[24] van der Made AD , Peters RW , Verheul C , Smithuis FF , Reurink G , Moen MH , et al. Proximal hamstring tendon avulsions: comparable clinical outcomes of operative and non‐operative treatment at 1‐year follow‐up using a shared decision‐making model. Br J Sports Med. 2022;56(6):340–348. 34996751 10.1136/bjsports-2021-104588

[25] Mazza D , Annibaldi A , Monaco E , Carrozzo A , Princi G , Fenucci S , et al. Achilles tendon rupture in professional football player: an epidemiological study in European championship with a mid‐term follow‐up. Muscles Ligaments Tendons J. 2022;12(4):478–483.

[26] Mazza D , Viglietta E , Monaco E , Iorio R , Marzilli F , Princi G , et al. Impact of anterior cruciate ligament injury on European professional soccer players. Orthop J Sports Med. 2022;10(2):23259671221076865. 35224121 10.1177/23259671221076865 PMC8873562

[27] Morgans R , Ju W , Radnor J , Zmijewski P , Ryan B , Haslam C , et al. The positional demands of explosive actions in elite soccer: comparison of English Premier League and French Ligue 1. Biol Sport. 2025;42(1):81–87. 39758183 10.5114/biolsport.2025.139083 PMC11694201

[28] Nieto Torrejón L , Martínez‐Serrano A , Villalón JM , Alcaraz PE . Economic impact of muscle injury rate and hamstring strain injuries in professional football clubs. Evidence from LaLiga. PLoS One. 2024;19(6):e0301498. 38870170 10.1371/journal.pone.0301498 PMC11175487

[29] Oliveira‐Júnior O , Gabbett TJ , Bittencourt NFN , Quintão RC , Reis GF , Claudino JG , et al. Potential financial loss and risk factors for hamstring muscle injuries in elite male Brazilian soccer players: a season‐long prospective cohort pilot study. Front Sports Active Living. 2024;6:360452. 10.3389/fspor.2024.1360452 PMC11458431 39381257

[30] Plastow R , Kerkhoffs GMMJ , Wood D , Paton BM , Kayani B , Pollock N , et al. London International Consensus and Delphi study on hamstring injuries part 2: operative management. Br J Sports Med. 2023;57(5):266–277. 36650033 10.1136/bjsports-2021-105383

[31] Robles‐Palazón FJ , López‐Valenciano A , De Ste Croix M , Oliver JL , García‐Gómez A , Sainz de Baranda P , et al. Epidemiology of injuries in male and female youth football players: a systematic review and meta‐analysis. J Sport Health Sci. 2022;11(6):681–695. 34700052 10.1016/j.jshs.2021.10.002 PMC9729930

[32] Transfermarkt . Transfermarkt market value explained—how is it determined? Hamburg, Germany: Transfermarkt; 2021. Available from: https://www.transfermarkt.co.in/transfermarkt-market-value-explained-how-is-it-determined-/view/news/385100

[33] Whiteley R , Massey A , Gabbett T , Blanch P , Cameron M , Conlan G , et al. Match high‐speed running distances are often suppressed after return from hamstring strain injury in professional footballers. Sports Health. 2021;13(3):290–295. 33151808 10.1177/1941738120964456 PMC8079800

[34] Wood DG , Packham I , Trikha SP , Linklater J . Avulsion of the proximal hamstring origin. J Bone Joint Surg Am. 2008;90(11):2365–2374. 18978405 10.2106/JBJS.G.00685

[35] Yi Q , Groom R , Dai C , Liu H , Gómez Ruano MÁ . Differences in technical performance of players from ‘The Big Five’ European Football Leagues in the UEFA Champions League. Front Psychol. 2019;10:2738. 31866914 10.3389/fpsyg.2019.02738 PMC6908525

